# Perillaldehyde 1,2-epoxide Loaded SLN-Tailored mAb: Production, Physicochemical Characterization and In Vitro Cytotoxicity Profile in MCF-7 Cell Lines

**DOI:** 10.3390/pharmaceutics12020161

**Published:** 2020-02-16

**Authors:** Eliana B. Souto, Selma B. Souto, Aleksandra Zielinska, Alessandra Durazzo, Massimo Lucarini, Antonello Santini, Olaf K. Horbańczuk, Atanas G. Atanasov, Conrado Marques, Luciana N. Andrade, Amélia M. Silva, Patricia Severino

**Affiliations:** 1 Department of Pharmaceutical Technology, Faculty of Pharmacy (FFUC), University of Coimbra, Pólo das Ciências da Saúde, Azinhaga de Santa Comba, 3000-548 Coimbra, Portugal; zielinska-aleksandra@wp.pl; 2 CEB—Centre of Biological Engineering, University of Minho, Campus de Gualtar 4710-057 Braga, Portugal; 3 Department of Endocrinology, Hospital de São João, Alameda Prof. Hernâni Monteiro, 4200-319 Porto, Portugal; sbsouto.md@gmail.com; 4 CREA-Research Centre for Food and Nutrition, Via Ardeatina 546, 00178 Rome, Italy; alessandra.durazzo@crea.gov.it (A.D.); massimo.lucarini@crea.gov.it (M.L.); 5 Department of Pharmacy, University of Napoli Federico II, 80131 Napoli, Italy; 6 Department of Technique and Food Product Development, Warsaw University of Life Sciences (WULS-SGGW) 159c Nowoursynowska, 02-776 Warsaw, Poland; olaf_horbanczuk@sggw.pl; 7 Institute of Neurobiology, Bulgarian Academy of Sciences, 23 Acad. G. Bonchev str., 1113 Sofia, Bulgaria; atanas.atanasov@univie.ac.at; 8 Institute of Genetics and Animal Breeding, Polish Academy of Sciences, Jastrzębiec, 05-552 Magdalenka, Poland; 9 Department of Pharmacognosy, University of Vienna, Althanstraße 14, 1090 Vienna, Austria; 10 Ludwig Boltzmann Institute for Digital Health and Patient Safety, Medical University of Vienna, Spitalgasse 23, 1090 Vienna, Austria; 11 Laboratory of Nanotechnology and Nanomedicine (LNMED), Institute of Technology and Research (ITP), Av. Murilo Dantas 300, Aracaju 49010-390, Brazil; conrado.marques@souunit.com.br; 12 Industrial Biotechnology Program, University of Tiradentes (UNIT), Av. Murilo Dantas 300, Aracaju 49032-490, Brazil; 13 Tiradentes Institute, 150 Mt Vernon St, Dorchester, MA 02125, USA; 14 Laboratory of Nanotechnology and Nanomedicine, Institute of Technology and Research, Aracaju SE 49032-490, Brazil; luciana.nalone@hotmail.com; 15 School of Pharmacy, University Tiradentes, Aracaju SE 49032-490, Brazil; 16 School of Biology and Environment, University of Trás-os-Montes e Alto Douro (UTAD), Quinta de Prados, P-5001-801 Vila Real, Portugal; amsilva@utad.pt; 17 Centre for Research and Technology of Agro-Environmental and Biological Sciences (CITAB), University of Trás-os-Montes e Alto Douro (UTAD), P-5001-801 Vila Real, Portugal

**Keywords:** perillaldehyde 1,2-epoxide, Compritol ATO 888, cationic SLN, streptavidin adsorption, MCF-7 cells

## Abstract

We have developed a new cationic solid lipid nanoparticle (SLN) formulation, composed of Compritol ATO 888, poloxamer 188 and cetyltrimethylammonium bromide (CTAB), to load perillaldehyde 1,2-epoxide, and surface-tailored with a monoclonal antibody for site-specific targeting of human epithelial growth receptor 2 (HER2). Perillaldehyde 1,2-epoxide-loaded cationic SLN (c*Pa*-SLN), with a mean particle size (z-Ave) of 275.31 ± 4.78 nm and polydispersity index (PI) of 0.303 ± 0.081, were produced by high shear homogenization. An encapsulation efficiency of c*Pa*-SLN above 80% was achieved. The release of perillaldehyde 1,2-epoxide from cationic SLN followed the Korsemeyer–Peppas kinetic model, which is typically seen in nanoparticle formulations. The lipid peroxidation of c*Pa*-SLN was assessed by the capacity to produce thiobarbituric acid-reactive substances, while the antioxidant activity was determined by the capacity to scavenge the stable radical DPPH. The surface functionalization of c*Pa*-SLN with the antibody was done via streptavidin-biotin interaction, monitoring z-Ave, PI and ZP of the obtained assembly (c*Pa*-SLN-*S*_Ab_), as well as its stability in phosphate buffer. The effect of plain cationic SLN (c-SLN, monoterpene free), c*Pa*-SLN and c*Pa*-SLN-*S*_Ab_ onto the MCF-7 cell lines was evaluated in a concentration range from 0.01 to 0.1 mg/mL, confirming that streptavidin adsorption onto c*Pa*-SLN-*S*_Ab_ improved the cell viability in comparison to the cationic c*Pa*-SLN.

## 1. Introduction

The nonselective delivery of anticancer drugs to the tumor site remains a challenge in chemotherapy and is the reason for the serious side effects of the classical treatments. Nanoparticles have partially solved this limitation, by reducing the systemic distribution of anticancer drugs by passive targeting. Cationic nanoparticles with a net positive surface charge have been proposed to further enhance cellular interaction and increase the cellular uptake of the loaded drug [1,2,3,4,5,6]. Site-specific delivery can be achieved via active targeting by surface modifying, such as with antibodies, aptamers and other targeting moieties (e.g., transferrin, folate) tailored to specific receptors [7,8].

Traditional medicine has countless of examples of natural compounds with several health benefits. Essential oils are indeed a source of phytochemicals of pharmaceutical and nutraceutical interest, with monoterpenes being their main constituents [9]. Monoterpenes show antioxidant, antimicrobial, analgesic, anxiolytic and anticancer properties, with an increasing interest as a source of therapeutic alternatives [10,11,12,13]. Perillyl alcohol, a naturally occurring monoterpene found in the essential oils peppermint and lavender, has been widely studied [14], demonstrating effectiveness against a variety of human tumor cell lines [15,16,17]. The monoterpene showed cytotoxicity and antitumor activity in various experimental models, and has already reached clinical trials for cancer treatment [15,18]. The cytotoxicity of perillyl alcohol analogues, such as (-)-8,9-perillaldehyde epoxide, (-)-perillaldehyde, (+)-limonene 1,2-epoxide and (-)-8-hydroxycarvotanacetone, has also been thoroughly characterized [15]. The anti-tumoral activity of perillaldehyde 1,2-epoxide has also been described by Andrade et al. [15,19]. The aim of this study has been the loading of perillaldehyde 1,2-epoxide into cationic solid lipid nanoparticles (cSLN) for site-specific delivery to breast cancer cells. Solid lipid nanoparticles (SLN) have been selected as a delivery system due to their composition in biocompatible and biodegradable lipids, with a reduced risk of cyto/genotoxic events [3,20,21]. Furthermore, these particles can be produced with cationic lipids so that the positive charge can then be functionalized with a monoclonal antibody against human epithelial growth receptor 2 (HER2) [8].

## 2. Materials and Methods

### 2.1. Materials

Compritol ATO 888 (glycerol behenate) was obtained as a gift from Gattefosse (Saint-Priest, France), Poloxamer 188 (trade name: Kolliphor^®^ P188) was bought from BASF (Ludwigshafen, Germany), the ErbB2/HER-2 monoclonal antibody (CB11) was obtained from ThermoFisher Scientific (Wilmington, USA) and cetyltrimethylammonium bromide (CTAB) was purchased from Sigma (Sintra, Portugal). Perillaldehyde, 3-(4,5-dimethyl-2-thiazolyl)-2,5-diphenyl-2H-tetrazolium bromide (MTT), doxorubicin (purity > 98%), Trolox, thiobarbituric acid (TBA), butylated hydroxytoluene (BHT), dimethyl sulfoxide (DMSO), methanol, hexane, ethyl acetate, hydrogen peroxide (30%) and potassium hydroxide were purchased from Sigma Chemical Co. (St. Louis, MO, USA). Double-distilled water was used throughout the work, after filtration in a MiliQ system (Millipore, Merck KGaA, Darmstadt, Germany).

### 2.2. Synthesis of Perillaldehyde 1,2-epoxide

The synthesis of perillaldehyde 1,2-epoxide was carried out as described by Andrade et al. [19], who analyzed the product by infrared and ^1^H- and ^13^C-NMR [15]. Briefly, a solution of 7.5% (*m*/*v*) perillaldehyde in methanol was mixed with hydrogen peroxide (30%) in a 250 mL flask, and kept in an ice bath (0–4 °C), to which a volume of 5 mL of potassium hydroxide (0.5 g/mL) was added dropwise. The reaction medium was stirred for a period of four hours, after which it was removed from the ice bath and the aqueous phase was extracted by washing it with 50 mL of dichloromethane. The organic phase was washed twice with 50 mL double-distilled water, dried with anhydrous sodium sulfate and concentrated in an IKA rotary evaporator (Staufen, Germany). Purification was done in a silica gel column chromatography, using a mixture of hexane/ethyl acetate (9:1) as eluant. A yield of 77.8% was obtained for perillaldehyde 1,2-epoxide.

### 2.3. Production of Cationic Solid Lipid Nanoparticles (cSLN)

#### 2.3.1. Non-Functionalized cSLN

The production of cationic SLN (cSLN) was carried out by hot high-shear homogenization, as described by Souto et al. [8], using glycerol behenate as solid lipid and poloxamer 188 as surfactant. Compritol (glycerol behenate) [5.0% (*w*/*v*)] was melted at 80 °C and then dispersed in an aqueous solution composed of 0.25% (*w*/*v*) poloxamer 188 and 0.5% (*w*/*v*) CTAB, heated up at the same temperature to produce an emulsion under stirring at 8000 rpm for 10 min in an Ultra-Turrax (Ultra-Turrax ^®^, T25, IKA, Staufen, Germany). The obtained emulsion was diluted (2:1) in cold water, kept at 4 ± 0.5 °C and further processed at 5000 rpm for five more minutes. The obtained particles were transferred to siliconized glass vials and stored at 4 ± 0.5 °C for further studies. For the loading of cSLN with the synthesized perillaldehyde 1,2-epoxide (c*Pa*-SLN), nanoparticles were produced as described, by adding the drug [0.5% (*w*/*w*)] to the melted lipid [4.5% (*w*/*v*)] prior to emulsification. Weightings were done in an analytical balance (Mettler Toledo, Giessen, Germany) with a readability of 0.005 mg.

#### 2.3.2. mAb-Functionalized cSLN

The functionalization of c*Pa*-SLN was carried out as previously described, and following the method proposed by Petersen et al. [22]. Firstly, the ability of the produced cationic nanoparticles to bind streptavidin was evaluated by incubating c*Pa*-SLN with the protein at decreasing ratios (1:5, 1:10, 1:15, 1:20 and 1:25), for a period of one hour at room temperature. The formation of the c*Pa*-SLN-Streptavidin (c*Pa*-SLN-*S*) complexes was monitored by determining z-AVE and ZP, as described in 2.4. The monoclonal antibody (mAb, CB11) was dispersed in PBS (pH 7.4), diluted down to 1 mg/mL and biotinylated using a Biotinylation Kit (Biotin Conjugation Kit (Fast, Type A) Lightning-Link^®^). Aliquots of biotinylated antibody were stored at −20 °C until further use. c*Pa*-SLN-*S* complexes were mixed with a biotinylated antibody and incubated at room temperature over at least one hour to complex with the mAb, and form c*Pa*-SLN-*S*_Ab_ complexes. The formation of the c*Pa*-SLN-*S*_Ab_ complexes (i.e., the adsorption of mAb onto the c*Pa*-SLN-*S* surface) was monitored by measuring z-AVE and ZP, as described in Section 2.4.

### 2.4. Mean Particle Size, Polydispersity Index and Zeta Potential

Immediately after the production of each nanoparticle batch, the mean particle size (z-Ave) and polydispersity index (PI) were determined by dynamic light scattering (DLS, Zetasizer Nano ZS, Malvern, Worcestershire, UK). Prior to the analysis of cSLN and c*Pa*-SLN, particles were diluted with MilliQ water and measured at a 1 mg/mL of solid lipid concentration. Prior to the analysis of c*Pa*-SLN-*S* and c*Pa*-SLN-*S*_Ab_, particles were diluted in a phosphate buffer saline (PBS, pH 7.4) and measured at a 1 mg/mL of solid lipid concentration. Zeta potential (ZP) was recorded in a laser Doppler anemometry Zetasizer Nano ZS (Malvern, Worcestershire, UK) using the Smoluchowski equation. Dilutions were performed prior to analysis, as described for the recording of z-Ave and PI. Measurements were done in triplicate (*n* = 3) (10 runs per measurement, 30 in total), and data were expressed as the arithmetical mean ± standard deviation (SD).

### 2.5. Encapsulation Efficiency (EE)

The encapsulation efficiency (EE) of perillaldehyde 1,2-epoxide in c*Pa*-SLN was determined as an indirect measure of the amount of drug quantified in supernatant [23]. Briefly, c*Pa*-SLN was firstly ultra-centrifuged for 1 h at 100,000 g in a Beckman Optima™ Ultracentrifuge (Optima™ XL, Indianapolis, IN, USA) and the quantification of perillaldehyde 1,2-epoxide, determined in the supernatant in a UV spectrophotometer Shimadzu UV-1601 (Shimadzu Italy, Cornaredo, Italy), at 245 nm. The following equation was used to calculate *EE*% [24]

(1) $$EE\%=\frac{W_{Pa}-W_{s}}{W_{Pa}}\times 100$$

where *W_PA_* is the mass of perillaldehyde 1,2-epoxide used for the production of SLN, and *W_S_* is the mass of perillaldehyde 1,2-epoxide quantified in the supernatant.

### 2.6. In Vitro Release Profile of cPa-SLN

Vertical Franz diffusion cells were used to determine the in vitro release profile of perillaldehyde 1,2-epoxide from c*Pa*-SLN. Prior to the assay, cellulose membranes with an average pore size of 0.22 µm (MERCK KgaA, Darmstadt, Germany) were firstly soaked for 2 h in PBS (pH 7.4), and then placed between the donor and acceptor compartments. A volume of 1 mL of freshly prepared c*Pa*-SLN was placed onto the top of the donor compartment. The acceptor compartment, containing 5 mL of a PBS buffer, was kept under magnetic stirring at 37 °C over the course of the assay. At pre-determined time intervals, a volume of 200 µL was sampled with a syringe, being the same volume replaced with the PBS buffer to ensure sink conditions. The cumulative amount of perillaldehyde 1,2-epoxide was analysed in a UV spectrophotometer Shimadzu UV-1601 (Shimadzu Italy, Cornaredo, Italy) at 245 nm. Four kinetic models, namely the zero order, first order, Higuchi and Korsmeyer-Peppas models, have been used for the mathematical fitting of the recorded values [25]. The obtained R^2^ values were used for the selection of the most appropriate model.

### 2.7. In Vitro Lipid Peroxidation Assay

To 1 mL of egg yolk homogenate (1% *w*/*v*) in phosphate buffer (pH 7.4), a volume of 0.1 mL ferrous sulphate (FeSO_4_, 0.17 mol/L) was added. To the obtained mixture, increasing concentrations of c*Pa*-SLN (1, 2, 3, 4, 5 and 10 µg/mL, solid lipid) were added, which were then incubated at 37 °C for 30 min. After cooling, a volume of 0.5 mL of each mixture was centrifuged with 0.5 mL of trichloroacetic acid solution (15% *m*/*v*) for 10 min at 1200 rpm. The collected supernatant (0.5 mL) was mixed with the same volume of thiobarbituric acid solution (0.67% *m*/*v*) and incubated for 60 min at 95 °C. After cooling, the formation of TBARS was quantified by spectrophotometry by measuring the supernatant at 532 nm, and the results were expressed as malondialdehyde equivalents (MDA Eq) of the substrate. Trolox (standard antioxidant) was used as positive control, at 50 μg/mL, against water as the negative control.

### 2.8. In Vitro Antioxidant Activity of cPa-SLN

The antioxidant activity of c*Pa*-SLN was determined as the ability of the loaded drug to scavenge the stable radical DPPH^•^ [26]. Briefly, c*Pa*-SLN was firstly dissolved in 0.1 mM of a DPPH methanolic solution to achieve concentrations of c*Pa*-SLN of 1, 2, 3, 4, 5 and 10 µg/mL of solid lipid. Then, 20 µL of samples were placed in the microplate wells. Finally, 200 µL DPPH methanolic solution (0.1 mM), were added to each of the wells. Methanol and butylated hydroxytoluene (BHT, 0–6 µg/mL) were used as negative and positive controls, respectively. The microplates were incubated at 25 °C for 30 min, and then read at 517 nm in a multiplate reader (DTX 880 Multimode Detector, Beckman Coulter Inc.). The antioxidant activity (AA) as the measure of the percentage of scavenging of free radicals was calculated from the recorded optical densities (OD), using the following equation:

(2) $$AA\%=\frac{\mathrm{OD}\;\mathrm{of}\;\mathrm{negative}\;\mathrm{control}\;-\mathrm{OD}\;\mathrm{of}\;\mathrm{sample}}{\mathrm{OD}\;\mathrm{of}\;\mathrm{negative}\;\mathrm{control}}\;\times 100$$

By plotting the concentration in the *X*-axis (μg/mL) against *AA%* in the *Y*-axis (% inhibition), the linear regression equation was obtained and the IC_50_ value determined.

### 2.9. Cell Culture and MTT Assay

The cytotoxicity of cSLN (blank) and c*Pa*-SLN was tested in MCF-7 cells obtained from ATCC (Pensabio Biotecnologia, São Paulo, Brazil). Cells were cultured in RPMI-1640 medium supplemented with 10% fetal bovine serum, 2 mM L-glutamine, 100 µg/mL streptomycin and 100 U/mL penicillin, and further incubated at 37 °C in a 5% CO_2_ atmosphere. Consumables for cell culture were obtained from Sigma Chemical Co. (St. Louis, MO, USA). For the 3-(4,5-dimethyl-2-thiazolyl)-2,5-diphenyl-2H-tetrazolium bromide (MTT) assay [27], cells were incubated in 96-well plates (0.1 × 10^6^ cells/mL; 100 μL/well) for 24 h. Solutions of cSLN (blank) and c*Pa*-SLN in dimethyl sulfoxide (DMSO 0.7%) at increasing concentrations (1, 2, 3, 4, 5 and 10 µg/mL of solid lipid) were added to each well, and incubated for more 72 h at 37 °C in a 5% CO_2_ atmosphere. A solution of DMSO 1% was set as the negative control, whereas a doxorubicin solution (100 μg/mL) was set as the positive control. At the end of the incubation period, test solutions were removed. An MTT solution (150 μL) at 0.5 mg/mL was added to each well, and incubated for three hours at 37 °C in a 5% CO_2_ atmosphere. Cell viability was determined as the ability of viable cells to reduce the yellow dye MTT to the purple formazan. The obtained precipitate was dissolved in 150 μL DMSO, and the absorbance was read at 595 nm using a multiplate reader (DTX 880 Multimode Detector, Beckman Coulter Inc.). The results were expressed as percentage of cell growth inhibition (%GI) as follows:

(3) $$\%GI=100\times \left[\frac{{\mathrm{Abs}}_{\mathrm{Test}}}{{\mathrm{Abs}}_{\mathrm{Negative}\;\mathrm{Control}}}\times 100\right]$$

### 2.10. Statistical Analysis

Data obtained are expressed as the mean ± SEM, and the differences among experimental groups were evaluated using a one-way analysis of variance (ANOVA) followed by the Dunnet post-test. Values of *p* < 0.05 were considered significant. All statistical analyses were carried using the GraphPad program 5.0^®^ (Intuitive Software for Science, San Diego, CA, USA).

## 3. Results and Discussion

From the *p*-menthane derivatives described by Andrade et al. [15], perillaldehyde 1,2-epoxide was selected due to its high cytotoxic profile (growth inhibition (*GI*%) > 95%), and was tested in a concentration of 25 μg/mL in colon carcinoma (HCT-116), ovarian adenocarcinoma (OVCAR-8), glioblastoma (SF-295) and promyelocytic leucemia (HL-60) cell lines [19]. Literature states that *GI*% = 0 means no cytotoxicity, while 1 < *GI*% < 50 is low cytotoxicity, 51 < *GI*% < 75 is moderate cytotoxicity and *GI*% > 75 is cytotoxicity [28]. To reduce the cytotoxicity of the compound while increasing site-specific delivery, we proposed the loading of perillaldehyde 1,2-epoxide into cationic solid lipid nanoparticles (SLN), to be surface tailored to HER2 receptors. The loading of the selected monoterpene into Compritol cSLN resulted in particles with the characteristics summarized in Table 1. The high-shear homogenization method has been previously shown to produce SLN of a low mean size and polydispersity [8], and the possibility to operate at a temperature compatible with the thermal stability of the selected drug [15,29].

The nonsurface modified cationic SLN showed a very high positive net charge in both batches, due to the presence of CTAB (0.5% *m*/*v*) on the surface. Both z-Ave and PI increased with the loading of the monoterpene, showing a slightly broad distribution with a PI above 0.24 (values below this limit are considered monodispersed). A slight decrease of ZP was found with the loading of perillaldehyde 1,2-epoxide, attributed to its lipophilic character, and confirming its loading within the lipid matrices.

**Figure 1:**
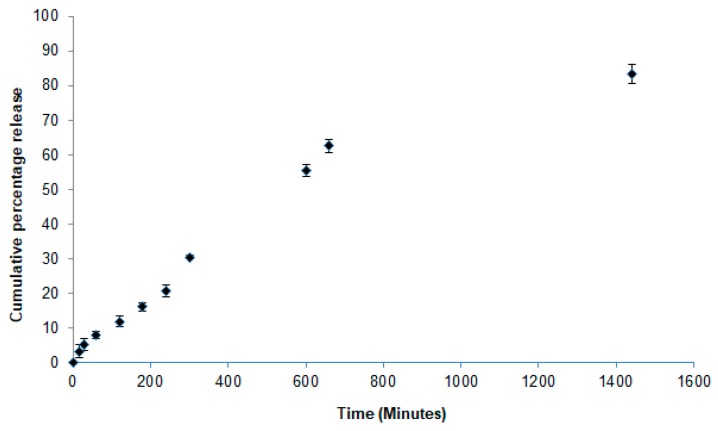
Cumulative percentage release of perillaldehyde 1,2-epoxide from *cPa*-SLN over 24 h.

Due to its lipophilic character, more than 80% of the drug was encapsulated within Compritol matrices. As SLN are of a crystalline nature, it is expected that a modified release profile can be achieved for the loaded drug. The release profile of *cPa*-SLN was evaluated over the course of 24 h, and the results are shown in Figure 1.

**Figure 2:**
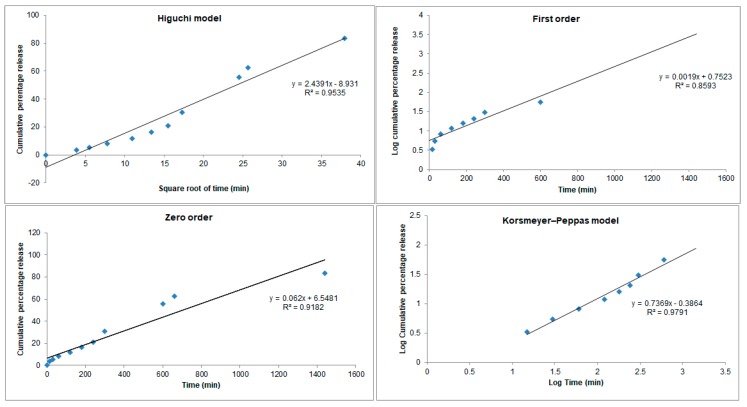
Mathematical fitting models of the release profile of perillaldehyde 1,2-epoxide from *cPa*-SLN over 24 h.

About 12% of the drug was released within the first two hours (11.90 ± 1.52%), while at the end of the 24-h period, 83.40 ± 2.79% of the drug was released. The depicted profile *cPa*-SLN translates a controlled release of perillaldehyde 1,2-epoxide from the cationic particles. To further elucidate which mechanisms are behind such releases, four mathematical models were used to fit the recorded values (Figure 2).

From the values obtained for *R*^2^, the best model describing the release of perillaldehyde 1,2-epoxide from *cPa*-SLN was shown to be Korsmeyers–Peppas, with a *R*^2^ of 0.9791, the closest straight-line results. This model describes the drug release from the nanoparticles accordingly to $M_{t}/M_{\infty }\;=\;k^{\prime }t^{n}$, where $M_{t}\;$is the cumulative amount of the drug released at time t, $M_{\infty }$ is the cumulative amount of the drug released at an infinite time, $k^{\prime }$ is the constant that is governed by the physicochemical properties of the nanoparticle matrix and *n* is the diffusional release exponent indicating of the mechanism of the drug release. Indeed, *n* = 0.5 stands for Fickian diffusion, whereas 0.5 < *n* < 1.0 means a non-Fickian diffusion. The shape of the particles plays a significant role on the drug release. For particles of a spherical shape, the drug release becomes independent of time and reaches a zero-order release, known as Case II transport, achieved as *n* approaches 1.0. In such cases, a diffusional exponent n = 1.0 is indicative of non-Fickian transport. If *n* > 1.0, super Case II transport is followed [30]. The second-best fitting model was Higuchi, with a *R*^2^ of 0.9535. The Higuchi model describes the fraction of the drug released from a matrix being proportional to the square root of time, i.e., Mt/M∞ =kH t12, where $M_{t}\;$is the cumulative amount of the drug released at time t, $M_{\infty }$ is the cumulative amount of the drug released at an infinite time, and $k_{H\;}$ is the Higuchi dissolution constant, which is governed by the physicochemical properties of the nanoparticle matrix. If the release profile follows this model (Fickian diffusion), it means that a straight line with $k_{H\;}$as a slope will be obtained when plotting *x* = $k_{H\;}$against *y* =$\;M_{t}/M_{\infty }$. The modified release profile is achieved because of the solid state of the lipid core, as previously confirmed by us [2]. Besides, we have also confirmed by DSC and x-Ray diffraction that cationic surfactant CTAB forms a stabilizing layer on the SLN surface, and is not part of the inner matrix, solely composed of solid lipid and the drug. These results were confirmed by the decrease of ZP over storage time, which means that CTAB may suffer some adsorption from the surface during its shelf life [31].

**Figure 3:**
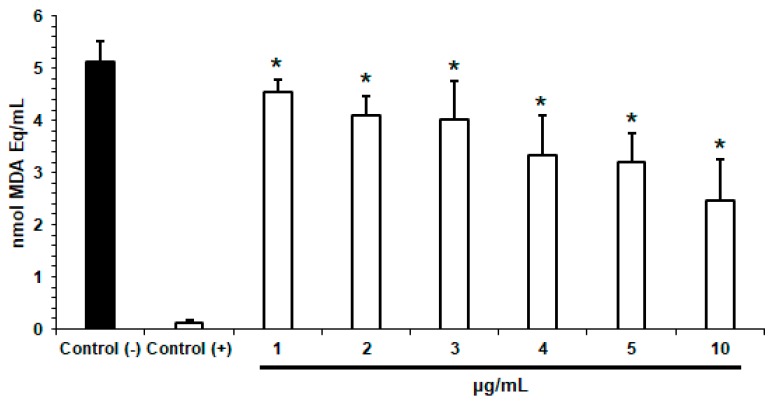
Effect of c*Pa*-SLN (1, 2, 3, 4, 5 and 10 µg/mL) on the amount of malondialdehyde equivalents (MDA Eq.) produced in the presence of the free radical FeSO_4_ inducers, performed in triplicate. Trolox and water were used as the positive and the negative control, respectively. Data are presented as mean ± SEM. * *p* < 0.05 when compared to the negative. One-way ANOVA with Dunnet post-test was applied.

Due to the vulnerability of lipid materials to free radicals, SLN can suffer lipid peroxidation. However, it is estimated that perillaldehyde 1,2-epoxide, as a monoterpene derivative, can neutralize the free radicals eventually resulting from lipid peroxidation. Increasing concentrations of c*Pa*-SLN (1, 2, 3, 4, 5 and 10 µg/mL) were assayed, and the results are depicted in Figure 3. As shown in Figure 3, the increasing concentration of the particles increased the neutralizing capacity attributed to the higher amount of the drug available to reduce the product formation generated by lipid peroxidation, i.e., the MDA (nmol MDA Eq/mL), when compared to the negative control (*p* < 0.05). The six tested concentrations (1, 2, 3, 4, 5 and 10 µg/mL) revealed an antioxidant effect, i.e., the capacity of c*Pa*-SLN to inhibit the Fenton reaction. This property is also linked to the capacity of terpenes in preventing DNA damage by neutralizing reactive oxygen species (ROS), widely reported as a major cause of cancer [32]. The capacity of c*Pa*-SLN to neutralize ROS was also confirmed using the DPPH test, and as expected, was shown to be concentration-dependent (Table 2). The absorbance decay of the sample test was correlated with the absorbance decay of the control test, resulting in the percentage scavenging of free radicals translated as the antioxidant activity [23]. For the positive control (BHT), 78.11% scavenging of DPPH radicals was recorded at the highest-tested concentration (6.0 µg/mL); similar results were previously reported [23,33]. By plotting the obtained results, a linear regression (*y* = 3.9814 *x* − 3.9867) with *R*^2^ = 0.9856 was obtained, and the IC_50_ was calculated as 195.08 µg/mL.

From the results depicted in Table 2, increasing the concentration of lipid nanoparticles, the amount of the viable drug also increases, considering that more than 80% of the drug is loaded in the lipid matrices and is released in a time-dependent fashion (Figure 2). Our results confirm that c*Pa*-SLN shows some antioxidant capacity (even if used at low concentration of particles) that can be exploited together with the antitumoral activity of perillaldehyde 1,2-epoxide in site-specific delivery. For a successful active targeting and site-specific delivery, the surface-modification of the particles is needed. The first step has been the streptavidin binding into c*Pa*-SLN (c*Pa*-SLN-*S*). Streptavidin is a protein purified from *Streptomyces* *avidinii*, showing high affinity for biotin, and is highly resistant to temperature variations, extreme pH values, organic solvents and proteolytic enzymes. It is usually recommended for the displaying of immobilized biotinylated antibodies [34,35].

**Figure 4:**
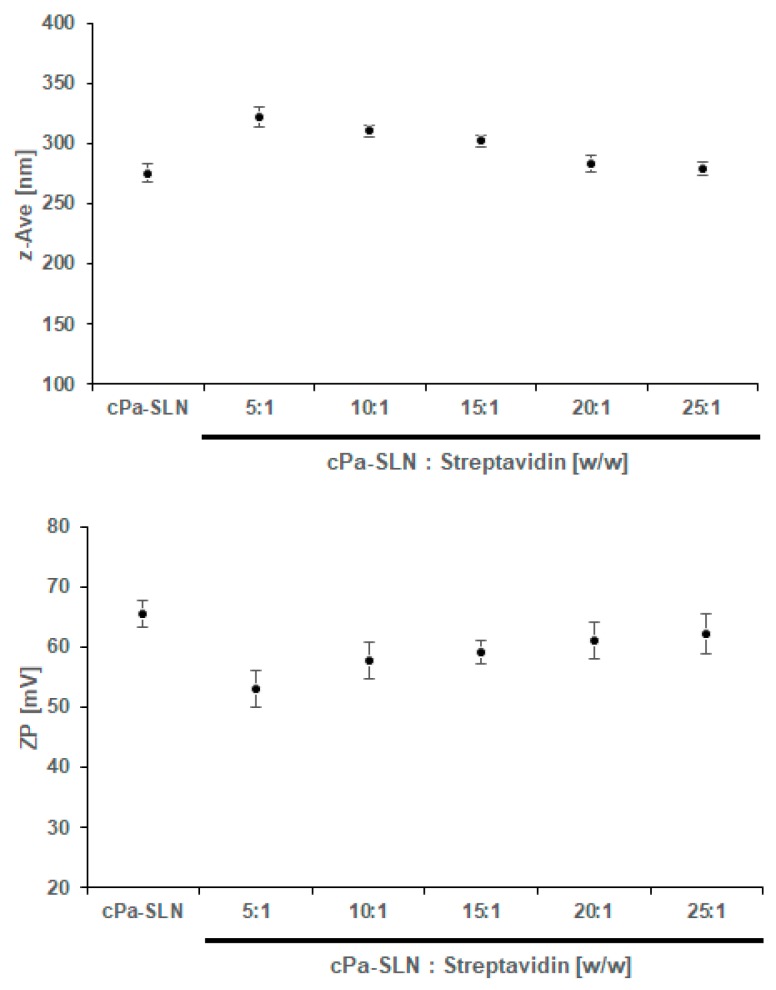
Variation on the mean particle size (z-Ave, upper panel) and zeta potential (ZP, lower panel) of c*Pa*-SLN-*S* complexes obtained from different c*Pa*-SLN—Streptavidin binding ratios. Results are given as a mean from three measurements of three independent experiments.

To evaluate the capacity of c*Pa*-SLN to bind streptavidin and produce the c*Pa*-SLN-*S* complex, c*Pa*-SLN were first diluted with PBS (1 mg/mL) and mixed, in different ratios, with aqueous streptavidin solution, as described by us [8], following the monitoring of z-Ave and ZP (Figure 4). While the amount of mAb successfully attached to biotin and then to the surface of cationic SLN could not be directly quantified, the amount of streptavidin and biotinylated antibody was optimized by a stepwise monitoring of the z-Ave and ZP of the obtained complexes, as well as their immediate stability in PBS.

**Figure 5:**
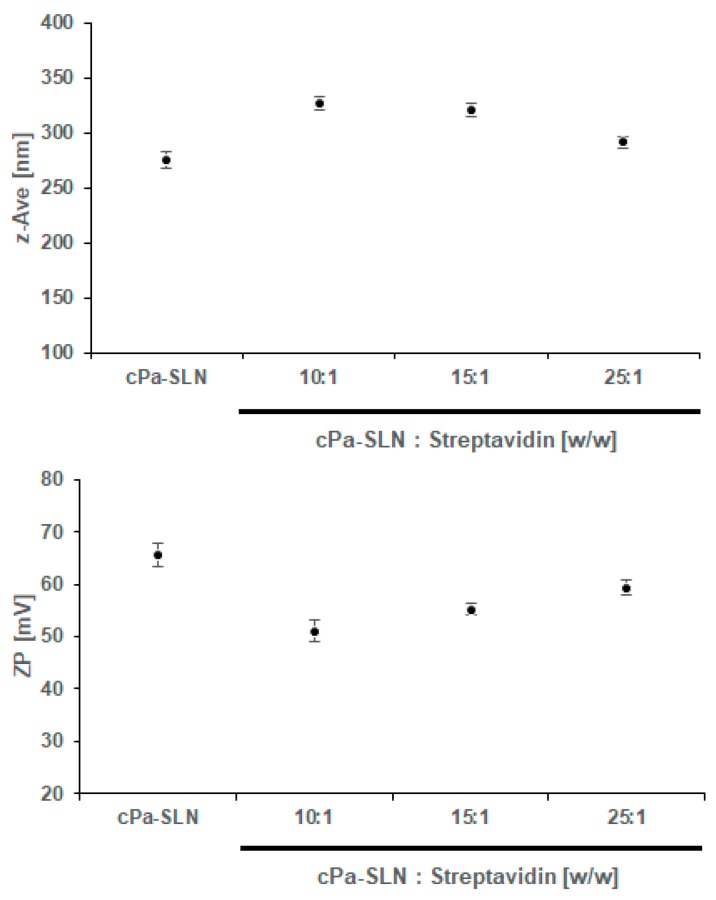
Variation on the mean particle size (z-Ave, upper panel) and zeta potential (ZP, lower panel) of c*Pa*-SLN-*S*_Ab_ complexes obtained from the binding of the antibody with different c*Pa*-SLN—Streptavidin binding ratios. Results are given as mean from three measurements of three independent experiments.

Although not statistically significant, a stepwise decrease in the z-AVE was shown with the increasing ratio of c*Pa*-SLN—Streptavidin, i.e., the higher the amount of protein bound to the surface the higher the particle size (Figure 4, upper). The ZP decreased from 65.57 ± 2.23 mV (c*Pa*-SLN) down to 53.06 ± 3.08 mV (5:1 *w*/*w*), which means that a stepwise decrease in the ZP was shown with the increasing amount of streptavidin. Although the decrease of ZP is associated with the increased risk of aggregation of particles in dispersion, the values remained well above +50 mV, ensuring a sufficient number of repulsive forces to maintain the electrostatic stability of the dispersions. Our results confirm the binding capacity of c*Pa*-SLN to streptavidin. To further check the binding of the obtained complexes with the monoclonal antibody (c*Pa*-SLN-*S*_Ab_), 10 µg of biotinylated mAb was mixed with c*Pa*-SLN-*S* complexes, obtained with ratios of 25:1, 15:1 and 10:1 in PBS. The z-Ave and ZP were again monitored (Figure 5).

The further increase in z-Ave with the antibody attachment up to 327.33 ± 6.21 nm and decreasing of ZP down to 51.04 ± 6.21 mV confirmed the binding and formation of c*Pa*-SLN-*S*_Ab_ complexes. Again, the high ZP values ensure their stability in aqueous dispersion. Besides, proteins adsorbed onto the nanoparticles’ surface can also provide some stereochemical stabilization, which was confirmed as no phase separation was seen.

**Figure 6:**
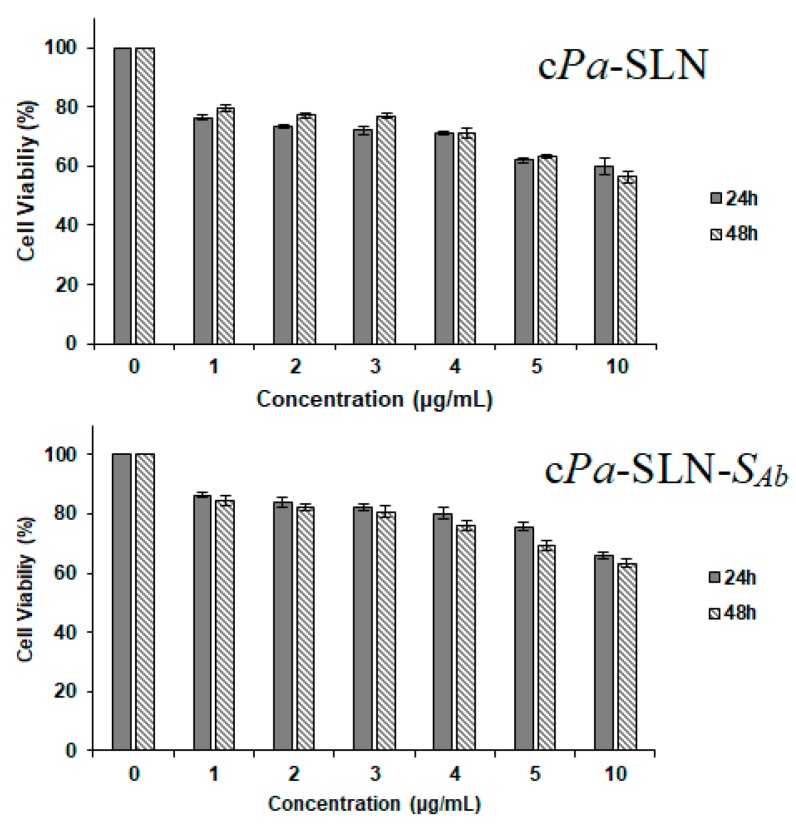
Evaluation of the cytotoxic activity of c*Pa*-SLN and c*Pa*-SLN-*S*_Ab_ in MCF-7 cell line using the MTT assay at 24 and 48 h.

For their further use as carriers in chemotherapy, the cytotoxicity of c*Pa*-SLN-*S*_Ab_ (10:1 ratio) was checked in comparison to the non-surface modified particles (c*Pa*-SLN) in MCF-7 cells (Figure 6). The cytotoxicity assay confirmed that the surface modification of the particles as the effect of the cationic lipid was attenuated, as shown by the increase in cell viability from 56.33 ± 1.99% when treated with c*Pa*-SLN, to 63.30 ± 1.45% when treated with c*Pa*-SLN-*S*_Ab_, at the highest-tested concentration. We also observed that, at the lowest concentration, a drop of about 20% in cell viability occurred. This effect was attributed to the presence of CTAB in the formulations [36].

## 4. Conclusions

The present study showed that the cytotoxic effect of perillaldehyde 1,2-epoxide against MCF-7 cell lines could be ameliorated when surface-modifying the particles with streptavidin. The particles exhibited some antioxidant capacity, attributed to the encapsulated monoterpene derivative. The cationic character of these particles provided a binding pathway via streptavidin to monoclonal antibody. The particles showed a modified release profile following the Korsemeyer–Peppas mathematical fitting. To further evaluate the affinity of mAb to HER2 receptors, the assessment of the targeting potential of the developed complexes and their cell internalization is planned, together with in vivo studies in a suitable animal model.

## Figures and Tables

**Table 1:** Mean particle size (z-AVE), polydispersity index (PI), zeta potential (ZP) and encapsulation efficiency (*EE*%) of perillaldehyde 1,2-epoxide into cationic SLN.

Batch	z-Ave (nm)	PI	ZP (mV)	EE%
cSLN	217.89 ± 5.33	0.293 ± 0.049	+67.91 ± 3.41	−
c*Pa*-SLN	275.31 ± 4.76	0.303 ± 0.081	+65.57 ± 2.23	81.64 ± 1.06

**Table 2:** Evaluation of antioxidant activity (% scavenging of free radical DPPH) of perillaldehyde 1,2-epoxide from c*Pa*-SLN.

µg/mL	AA%
1	0.59 ± 0.03
2	4.24 ± 0.02
3	7.39 ± 0.10
4	11.27 ± 0.05
5	14.93 ± 0.11
10	21.27 ± 0.12
